# Odor identification performance in children aged 3–6 years

**DOI:** 10.1038/s41390-020-1083-3

**Published:** 2020-07-26

**Authors:** Valentin A. Schriever, Liesa Zscheile, Janine Gellrich, Thomas Hummel

**Affiliations:** 1 Abteilung Neuropädiatrie, Klinik und Poliklinik für Kinder- und Jugendmedizin, Medizinische Fakultät Carl Gustav Carus, Technische Universität, Dresden, Germany; 2 Smell and Taste Clinic, Department of Otorhinolaryngology, Medizinische Fakultät Carl Gustav Carus, Technische Universität, Dresden, Germany

## Abstract

**Background:**

While valid and reliable olfactory tests have been developed for children aged >5 years, olfactory testing has not systematically been evaluated in younger children. The aim of this study was to evaluate the reliability and validity of the “U-Sniff” odor identification test in children aged 3–6 years.

**Methods:**

We included 160 healthy children (age range 3–6 years) and 14 congenitally anosmic children. Participants were investigated in two identical sessions. The “U-Sniff” test was used to evaluate olfactory function. A picture identification test (PIT) and the Kasel-Concentration-Task (KKA) were administered to identify factors influencing odor identification performance.

**Results:**

Age significantly influenced odor identification performance, with older children achieving higher scores. PIT and KKA scores correlated positively with odor identification scores. The “U-Sniff” test demonstrated a high test–retest reliability (*r*_160_ = 0.75, *p* < 0.001). It was possible to distinguish between healthy and anosmic children by means of “U-Sniff” scores starting at age 4 years with high sensitivity (79–93%) and specificity (88–95%).

**Conclusions:**

The “U-Sniff” test is feasible for children starting at age 3 years. In children aged ≥4 years, it is a reliable and valid method to distinguish between normal olfactory function and anosmia.

**Impact:**

- Olfactory testing is reliable and valid starting at an age of 4 years.
- The study adds a systematic evaluation of olfactory testing in young children.
- Results of this study are especially interesting for clinicians in the diagnosis of olfactory dysfunction.

## Introduction

In a clinical setting, olfactory assessment often has to distinguish between a normal and a reduced sense of smell. To this end, several tests are available. The most frequently used tests are the University of Pennsylvania Smell Identification Test (UPSIT) and the “Sniffin’ Sticks” test battery.^1,2^ Using olfactory threshold tests, previous studies have shown that olfactory function in children is comparable to olfactory sensitivity in adults.^3–5^ These tests are lengthy and therefore difficult to perform in a young pediatric population, especially in demanding clinical settings. Therefore, odor identification tests have been developed considering the special requirements of children as specified by Dalton et al.^6^ This resulted in the development of, for example, the “Smell Wheel,”^7^ the “Sydney Children’s Hospital Odor Identification Test” (SCHOT),^8^ the “U-Sniff” odor identification test,^9^ or the NIH-Toolbox.^10^

Scores from most odor identification tests are age dependent with older children performing better than younger children^11–13^ (for a review, see ref. ^14^). Factors that might contribute to this increase in odor identification performance with age include linguistic development^5,15^ and odor familiarity.^7,12,16,17^ This leads to the question at which age olfactory testing can be reliably performed in children. Although several studies have included children starting at age 3 years,^11,12,17–20^ most of these studies have stated that reliable olfactory testing by means of odor identification ability is only possible in children aged >5 years, due to a high rate of test incompletion, large variability of test results, or poor odor identification.^11,17–19^ Although the correlation between test and retest has only been assessed in one study in 3–4-year-old children resulting in an *r* = 0.45,^10^ still other research proposed the feasibility of odor identification testing in children as young as 3 years of age.^7,12,20^

For a test to be clinically useful, it is mandatory to produce highly reliable and valid results. Hence, an odor identification test must have a high test–retest reliability, and it must distinguish between normosmia and reduced olfactory function. Although these requirements are met by the “U-Sniff” odor identification test for children aged ≥6 years,^9^ they have not been evaluated for an odor identification test for children aged <6 years.

Aim of this study was to evaluate the feasibility, reliability, and validity of the “U-Sniff” odor identification test in young children 3–6 years of age. In addition, factors influencing odor identification performance should be investigated. We hypothesized that odor identification performance and reliability of test results increase with age and that odor identification scores are related to cognitive function measured by means of picture identification and attention testing. Concerning test validity, we hypothesized that it is possible to distinguish between anosmic and healthy children with high sensitivity and specificity based on odor identification performance.

## Methods

### Ethics statement

This study received the approval of the local Ethics Committee. All aspects of the study were conducted in accordance with the Declaration of Helsinki on Biomedical Studies Involving Human Subjects. The purpose and the procedure of the study were explained to the children and their parents/legal guardians in verbal and written form. Children aged <8 years received a verbal explanation of the procedure. Written informed consent was obtained from parents/legal guardian prior to inclusion into the study. Every child gave his/her assent to participate in this study.

### Participants

Sample size estimates were performed using G*Power^21^ to detect differences in odor identification performance between four age groups ((group i) 3 years, (group ii) 4 years, (group iii) 5 years, and (group iv) 6 years) as well as examining the reliability of test performance. Estimates were based on previous studies using odor identification testing.^9,22^ Using an analysis of variance with alpha 0.05, a power of 0.9, and an effect size of 0.3, a sample size of *n* = 38 for each age group is necessary to detect group differences in odor identification performance. To evaluate the reliability of odor identification by means of a bivariate correlation analysis with alpha 0.05, a power of 0.9, and an expected correlation coefficient of *r* = 0.8, a sample size of *n* = 38 for each age group is needed. A total of *n* = 160 participants (*n* = 40 in each age group) were therefore included in the study.

Children were recruited in local kindergartens. A total of 160 healthy children (50% girls) with age ranging from 3 to 6 years (mean ± SD: 4.5 ± 1.1 years) were included in the study. Children reported normosmia and absence of disorders known to influence olfactory function.^23,24^ In addition, parents reported no abnormalities regarding their children’s sense of smell. All children finished the study, and no child had to be excluded from the analysis. Age of children did not differ between girls (4.5 ± 1.1 years) and boys (4.5 ± 1.1 years) (*t* = 0.0, *p* = 1.0). According to the age of children, four age groups were formed (*n* = 40 for each age group, 50% girls in each group): (i) 3 years, (ii) 4 years, (iii) 5 years, and (iv) 6 years.

In addition, olfactory test results of 14 children (8 girls, 6 boys, age 14.2 ± 3.1 years, range 6–17 years) with isolated congenital anosmia (ICA) were included for test validation. These children were previously tested with the original “Sniffin’ Sticks” test (olfactory threshold, odor discrimination, and 16-item odor identification) and were diagnosed as having ICA. All 14 children with ICA were also tested with the “U-Sniff” odor identification test.

### Procedure

The study consisted of two sessions, with a duration of approximately 25 min per session. The following tests were conducted during the first session after obtaining informed consent: picture identification test (PIT), odor identification test, and the Kasel-Concentration-Task for children aged 3–8 years. The same tests were performed during the second session with a mean interval of 7.28 ± 4.29 days (range 3–36 days).

### Picture identification test

The PIT was performed to control for cognitive and verbal function of the children. We used the 12 cards from the odor identification and 5 additional cards with items not included in the odor identification test. The test was performed in a four-alternative forced choice design. Children were asked to identify a target picture out of four presented pictures. The target was given verbally to the children. The order of target items was changed from the odor identification test. Only the 12 items of the PIT that were also included in the odor identification test were used for further analysis. The correct answers were summed up to the PIT score. The PIT score ranged from 0 to 12 points.

### Olfactory assessment

The “U-Sniff” odor identification test (Burghart GmbH, Wedel, Germany) was used to assess olfactory function.^9^ The “U-Sniff” is a 12-item odor identification test, based on the “Sniffin’ Sticks”, which has been developed for children. Normative data, obtained in a large population, are available for children aged 6–17 years.^22^ Each odor was presented separately to the children by removing the cap of the pen and positioning the pen approximately 2 cm under the participant’s nose for 3 s. The participants’ task was to identify the odor of each pen with the help of four descriptors. The descriptors were presented as pictures and in writing on flash cards. In addition, descriptors were read to the participants. The sum of correct answers was regarded as the odor identification score, which could range from 0 to 12 points.

### Kaseler-Konzentrations-Aufgabe (KKA; Kasel-Concentration-Task for children aged 3–8 years)

The KKA is a test to measure short-term attention and concentration ability in children aged 3–8 years. The task is to cross out target objects in rows of drawings. The time limit is set to 10 s per row. The score was converted into an age-dependent percentile rank. Normative data, based on a large population, are available for children aged 3–8 years.^25^

### Statistical analyses

For statistical analysis, the IBM SPSS 23.0 (SPSS Inc., Chicago, IL, USA) software was used. To analyze the potential factors on odor identification performance, generalized linear mixed models with Bonferroni post hoc tests were applied with the dependent variable “U-Sniff” odor identification score and the independent variables age (3, 4, 5, 6 years), sex (girls, boys), and session (first and second session). Pearson correlations were used to evaluate test–retest reliability of the “U-Sniff” odor identification test. A multiple linear regression was performed to analyze the possible effects of age, sex, PIT score, and KKA percentile rank on the “U-Sniff” odor identification score. Because of the nature of the underlying data, nonparametric tests were used whenever appropriate. Receiver operator characteristic curve (ROC) was used in conjunction with the Youden index (*Y* = sensitivity + specificity − 1)^26^ to define the cut-off value between normosmia and olfactory dysfunction with the highest sensitivity and specificity for each age group separately. In addition, positive predictive value (PPV) and negative predictive value (NPV) were calculated. The alpha level was set at *p* < 0.05.

## Results

### Olfactory test results

**Fig. 1:**
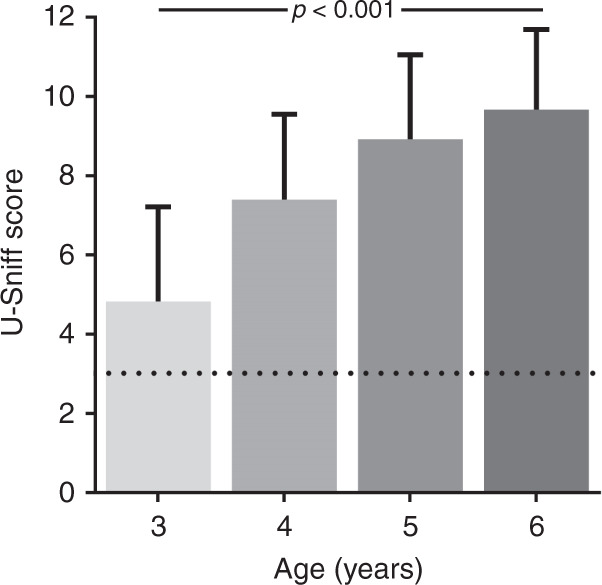
Odor identification score increases with age. Displayed are the “U-Sniff” odor identification scores for each of the four age groups (mean + SD). Odor identification scores significantly increase from age 3 to 6 years and differ significantly between all age groups. Dotted line indicates chance level.

At the first session, the mean odor identification score of the 160 children was 7.71 ± 2.85 points (range 1–12 points). Generalized linear mixed models were used to examine the effect of age, sex, and session on the odor identification score. A main effect of age (*F*_[df = 3]_ = 63.05, *p* < 0.001) was found, with younger children scoring lower on the odor identification test. Post hoc analyses revealed a significant difference between all age groups: (i) 5.23 ± 2.36 (range 1–10 points), (ii) 7.45 ± 2.36 (range 1–11 points), (iii) 8.79 ± 2.08 (range 1–12 points), and (iv) 9.72 ± 2.01 (range 3–12 points) (*t*s between 2.71 and 12.95, all *p*s < 0.01). For graphical display, see Fig. 1. Neither sex (girls: 7.91 ± 2.81 points, boys: 7.69 ± 2.74 points, *F*_[df = 1]_ = 0.72, *p* = 0.40) nor session (first: 7.71 ± 2.85 points, second: 7.89 ± 2.71 points, *F*_[df = 1]_ = 0.56, *p* = 0.45) had a significant effect on odor identification scores. In addition, no statistically significant interaction was observed between the factors sex and age (*F*_[df = 3]_ = 0.91, *p* = 0.44).

### PIT and KKA

Children scored on average 11.43 ± 0.98 points (range 6–12 points) on the PIT in the first session: They identified on average 95% of the pictures correctly. A main effect of age was seen (*F*_[df = 3]_ = 13.90, *p* < 0.001) with younger children scoring lower than older children (age group (i) 11.24 ± 0.88 points, (ii) 11.26 ± 1.24 points, (iii) 11.80 ± 0.43 points, and (iv) 11.90 ± 0.30 points). In addition, there was a main effect of session (*F*_[df = 1]_ = 7.11, *p* = 0.008) with higher scores obtained in the second session (11.67 ± 0.70 points) compared to the first session (11.43 ± 0.98). However, there was no main effect of the factor sex (*F*_[df = 1]_ = 0.67, *p* = 0.42).

On average, children scored a percentile rank of 63 ± 25 (range 1–100) on the KKA in the first session. Main effects were observed for age (*F*_[df = 3]_ = 15.03, *p* < 0.001, with older children achieving higher scores; age group (i) 52 ± 17, (ii) 62 ± 23, (iii) 74 ± 26, and (iv) 74 ± 30) and sex (*F*_[df = 1]_ = 13.83, *p* < 0.001) with girls (71 ± 25) reaching higher percentile rank compared to boys (61 ± 25), but no effect of the factor session (*F*_[df = 1]_ = 2.93, *p* = 0.088) was found.

### Factors influencing odor identification

**Fig. 2:**
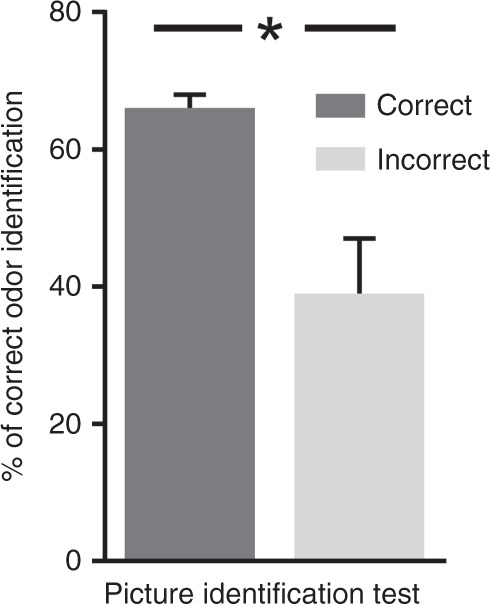
Influence of PIT on “U-Sniff”. Displayed are the percentages of correct odor identification scores for two conditions: correct item identification on the PIT (dark gray) and incorrect item identification on the PIT (light gray). Odor identification differed significantly between the two conditions. *p < 0.001.

A multiple linear regression was performed to determine the association between odor identification scores and the independent variables age, sex, PIT score, and KKA percentile rank, resulting in a significant model (*F*_[df = 3]_ = 59.39 *p* < 0.001) with an *R*^2^ = 0.423. Further analyses showed the independent effects of age (standardized coefficients are reported) (*β* = 0.486, *p* < 0.001), PIT score (*β* = 0.205, *p* < 0.001), and KKA percentile rank (*β* = 0.141, *p* = 0.003) but not sex (*β* = 0.019, *p* = 0.668) on odor identification performance. In addition, both PIT score (*r*_160_ = 0.41, *p* < 0.001) and KKA percentile rank (*r*_160_ = 0.35, *p* < 0.001) exhibited a positive correlation with the odor identification score. Further analysis was performed to observe the influence of incorrect identification of items on the PIT on the odor identification of the same items. Only 39% of odors were correctly identified when the same item was not identified on the PIT, whereas this number increased to 66% if items were identified correctly on the PIT (*z* = 6.70, *p* < 0.001, *d*_Cohen_ = 0.2; Fig. 2).

### Test reliability

**Fig. 3:**
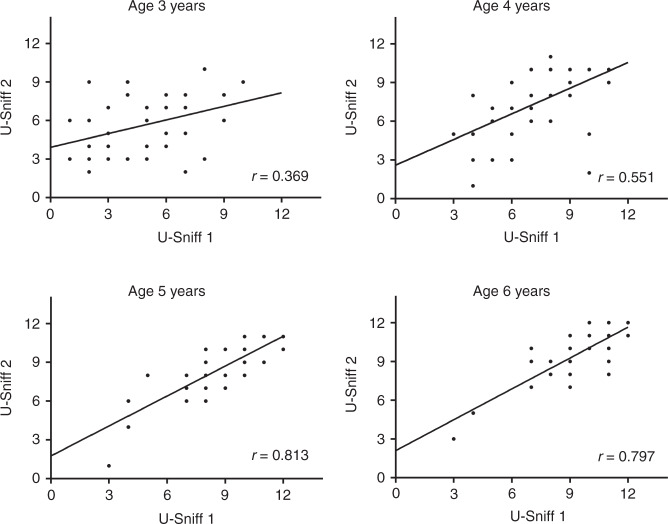
Reliability of “U-Sniff” odor identification test. Displayed are the correlation analyses of the “U-Sniff” odor identification test for each of the four age groups. Correlation coefficients increase with age and are significantly different between the two younger and two older age groups.

As reported above, there was no main effect of session on odor identification score. A strong positive correlation between odor identification scores from the first and second session was observed for the study population (*r*_160_ = 0.75, *p* < 0.001). This was also true for each age group separately (age group (i) *r*_40_ = 0.37, *p* = 0.019; (ii) *r*_40_ = 0.55, *p* < 0.001; (iii) *r*_40_ = 0.81, *p* < 0.001; and (iv) *r*_40_ = 0.80, *p* < 0.001; Fig. 3). Fisher transformation was used to build confidence intervals for the correlation coefficients. Significant differences in reliability coefficients were found between the two younger and two older age groups (age group (i) vs (iii): *z* = 3.11, *p* < 0.001; (i) vs (iv): *z* = 3.02, *p* < 0.001; (ii) vs (iii): *z* = 2.22, *p* = 0.013; and (ii) vs (iv): *z* = 2.02, *p* = 0.021).

### Test validation

**Fig. 4:**
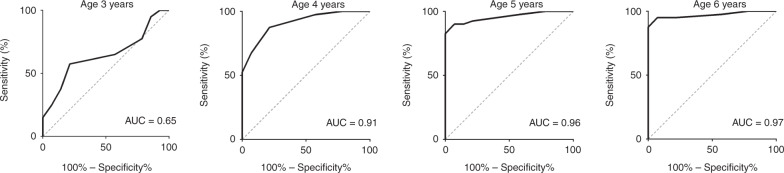
Receiver operating characteristic (ROC). Displayed are ROC analyses to distinguish between healthy children and children with ICA using odor identification test for each age group separately. With exception of the youngest age group, it was possible to distinguish between ICA and healthy children with high sensitivity and specificity. AUC area under the curve.

Children with ICA scored significantly lower on the “U-Sniff” odor identification test (3.57 ± 1.83 points) than healthy children (7.71 ± 2.85 points) (*t*_[df = 172]_ = 5.33, *p* < 0.001). The ROC analysis, performed for each age group separately, to distinguish between ICA and healthy children by means of the odor identification score showed an area under the curve (AUC) of 0.91 for children aged 4 years, 0.96 for 5 years, and 0.97 for 6 years (all *p*s < 0.001). It was not possible to distinguish between healthy children and ICA children by means of odor identification score in the youngest age group (AUC: 0.65, *p* = 0.110; Fig. 4). By using the highest Youden index to confirm olfactory dysfunction, a sensitivity and specificity of 79% and 88%, respectively, were reached when a cut-off of <5 points was used for 4-year-old children. For children aged 5 and 6 years, a sensitivity of 93% (both age groups) and specificity of 90% and 95%, respectively, to confirm olfactory dysfunction were reached when a cut-off of <7 points on the odor identification test was used. Based on these cut-off values, a PPV of 76.5% and an NPV of 97.3% for children aged 5 years and a PPV of 86.7% and an NPV of 97.6% for 6-year-old children resulted. For the younger age group of 4-year-old children, the PPV was 68.8% and the NPV was 92.1%.

## Discussion

In the current study, all 160 children aged 3–6 years were able to finish the study. Age significantly influenced odor identification performance. In addition, results of the PIT and KKA were positively correlated with odor identification. Data of this study indicated a good validity of the test in children aged ≥4 years.

Previous studies have shown that the “U-Sniff” odor identification test produces valid and reliable results in a wide age range of children starting at 6 years.^9,22^ The current study also shows the feasibility of the “U-Sniff” odor identification test in younger children starting at 3 years of age. All children, even in the youngest age group, finished the test. Incompletion of olfactory test performance in young children has been reported to be between 6 and 44% of children, depending on the test procedure and especially the length of olfactory testing in previous studies.^18,20,27^ The “U-Sniff” odor identification test was specifically developed for children, taking test length (12-items) and type of odors into account.^9^ Hugh et al. compared odor identification performance in children aged 5–12 years between the 40-item UPSIT and 12-item “Sniffin’ Sticks” odor identification tests and observed better performance in the shorter 12-item “Sniffin’ Sticks” test.^19^ This is in agreement with our present results and previous research suggesting that a short odor identification test is well suited for young children.^7,12^

In the present study, odor identification scores increased in children from 3 to 6 years. This is in agreement with previous work reporting an increase in odor identification performance with age in children, especially before adolescence.^7,11,22^ We included two cognitive tasks, the KKA and PIT, to control for attention and verbal function of the children. The KKA had an independent effect on odor identification score in this age group. Children scoring high on the attention and concentration ability measurement also reached higher odor identification scores. Still, although statistically significant, with a standardized coefficient of *β* = 0.141 the effect of KKA on odor identification score is small. This supports our previous statement that the “U-Sniff” odor identification test can be used in young, normally developed children to assess olfactory function.

Regression analysis showed an independent effect of the PIT score on odor identification performance. A PIT measures receptive verbal function and has been used in modified versions in previous studies.^3,6,11,17,20^ Positive correlations between the ability to identify pictures and odor identification scores have been reported.^11,17^ In our study, children reached an average higher score on the PIT (95% correct identification) than on the odor identification test (64% correct identification). This suggests that children are familiar with the items, which is in line with previous studies demonstrating similar results.^11,20^ Correct visual identification of an item is crucial for correct odor identification. In the current study, only 39% of odors were correctly identified when visual identification on the PIT was incorrect, whereas this number increased to 66% if items were identified correctly on the PIT. Semantic knowledge about an odor item is necessary for correct odor identification, which increases during childhood development.^5^ Cavazzana et al. assumed that in young children the cross-modal integration between odors and visual information about an odor item is not fully developed^18^ leading to better performance on the PIT compared to an odor identification test.

Although all children within the youngest age group were able to finish the odor identification test, the test–retest reliability was only moderate in this age group (*r* = 0.369). The reliability increased significantly with age with good (4-year-old children) and high test–retest reliability in children aged ≥5 years. This reliability is comparable to or higher than what has been reported for the majority of pediatric odor identification tests. It has to be kept in mind that the average age of our study population was younger than those of previous studies (“Sniffin Kids”: *r* = 0.44, NIH-Toolbox *r* = 0.45, “Smell Wheel”: *r* = 0.70, “SCHOT”: *r* = 0.98).^7,8,10,16^

By including 14 children diagnosed with ICA, the “U-Sniff” odor identification test was validated for young children. Because ICA is a rare condition in children, the age range in this population was set to <18 years to increase the number of children. Although previous and the current study have reported an increase in odor identification score with age,^7–9,11^ this increase is not expected in children with ICA. In fact, no correlation between age and odor identification scores was found in children with ICA in our study (*ρ* = −0.37, *p* = 0.194). Therefore, the difference in age range between the study populations should not affect the study outcome. Only few odor identification tests have been validated for a pediatric population by including anosmic individuals. Only for the “U-Sniff” odor identification test, cut-off values are available for children aged ≥6 years to distinguish between normosmia and olfactory dysfunction, which were based on ROC analysis.^9,16,17^ However, no odor identification test has been validated so far for children aged 3–6 years. Our study results show that it is possible to distinguish between children with ICA and healthy children with high sensitivity and specificity from age 5 years using the “U-Sniff” odor identification test when a cut-off value of <7 points is applied. The sensitivity (79%) and specificity (88%) to distinguish between healthy children and children with ICA in 4-year-old children was comparably lower. This has to be considered when evaluating olfactory function in children aged <5 years. The “U-Sniff” odor identification test failed to distinguish between healthy children and children with ICA in 3-year-old children. The results of odor identification testing in children in this age group is highly variable^17^ and might therefore not be suited for valid olfactory assessment.

Odor identification testing is a fast and reliable method for assessing olfactory function in adults and even very young children. This test is especially suitable for olfactory testing in clinical routine with limited time resources. Previous studies have also shown feasibility of the “Sniffin’ Sticks” olfactory threshold and odor discrimination test in children starting at age 5 years.^22,28^ Both olfactory threshold and odor discrimination testing is time-consuming and require a longer attention span, which might limit its use in very young children. Further studies need to address the question whether these tests are also suitable and produce reliable and valid results in olfactory assessment in younger children.

In conclusion, results of the current study show the feasibility of the “U-Sniff” odor identification test in children aged 3–6 years. Odor identification performance as well as reliability of the test increase with age. The “U-Sniff” odor identification test is a valid method to distinguish between normal olfactory function and anosmia in children aged ≥4 years. We therefore present an odor identification test, the “U-Sniff” test, which can be used in a clinical setting, resulting in valid and reliable results, starting at age 4 years.
