# Correction to: Odor identification performance in children aged 3–6 years

**DOI:** 10.1038/s41390-021-01675-4

**Published:** 2021-08-17

**Authors:** Valentin A. Schriever, Liesa Zscheile, Janine Gellrich, Thomas Hummel

**Affiliations:** 1 Abteilung Neuropädiatrie, Klinik und Poliklinik für Kinder- und Jugendmedizin, Medizinische Fakultät Carl Gustav Carus, Technische Universität, Dresden, Germany; 2 Smell and Taste Clinic, Department of Otorhinolaryngology, Medizinische Fakultät Carl Gustav Carus, Technische Universität, Dresden, Germany

Correction to: *Pediatric Research* 10.1038/s41390-020-1083-3, published online 26 July 2020

The article Odor identification performance in children aged 3–6 years, written by Valentin A. Schriever, Liesa Zscheile, Janine Gellrich, and Thomas Hummel was originally published Online First without Open Access. After publication in volume 89, issue 5, page 1304–1309, the author decided to opt for Open Choice and to make the article an Open Access publication. Therefore, the copyright of the article has been changed to © The Author(s) 2020 and the article is forthwith distributed under the terms of the Creative Commons Attribution 4.0 International License, which permits use, sharing, adaptation, distribution, and reproduction in any medium or format, as long as you give appropriate credit to the original author(s) and the source, provide a link to the Creative Commons licence, and indicate if changes were made.

The images or other third-party material in this article are included in the article’s Creative Commons licence, unless indicated otherwise in a credit line to the material. If material is not included in the article’s Creative Commons licence and your intended use is not permitted by statutory regulation or exceeds the permitted use, you will need to obtain permission directly from the copyright holder.

To view a copy of this licence, visit http://creativecommons.org/licenses/by/4.0/.

